# RBP4 and THBS2 are serum biomarkers for diagnosis of colorectal cancer

**DOI:** 10.18632/oncotarget.21173

**Published:** 2017-09-21

**Authors:** Weiqiang Fei, Li Chen, Jiaxin Chen, Qinglan Shi, Lumin Zhang, Shuiping Liu, Lingfei Li, Lili Zheng, Xiaotong Hu

**Affiliations:** ^1^ Biomedical Research Center and Key Laboratory of Biotherapy of Zhejiang Province, Sir Run Run Shaw Hospital, Zhejiang University, Hangzhou, China

**Keywords:** RBP4, THBS2, biomarker, colorectal cancer

## Abstract

The potential role of serum RBP4 and THBS2 as biomarker in colorectal cancer (CRC) diagnosis has never been studied. We investigated in large sample using quantitative ELISA method to explore whether serum RBP4 and THBS2 can act as biomarkers for CRC diagnosis. The concentration of RBP4 and THBS2 was measured in 402 CRC patients’ serum samples and 218 normal controls’ serum samples. The results showed that the average RBP4 and THBS2 concentrations in normal controls were significantly higher than in CRC patients (36.5±11.4μg/mL vs 21.8±8.7μg/mL and 20.5±6.1ng/mL vs 14.5±7.3ng/mL, respectively), both p<0.001. RBP4 distinguished CRC patients from normal individuals with the area under the receiver operating characteristic curve (AUC) performing at 0.852, with sensitivity of 74.9% and specificity of 81.7%. While THBS2 distinguished CRC patients performing AUC at 0.794, with sensitivity of 64.9% and specificity of 87.1%. The ability of RBP4 and THBS2 serum concentration distinguishing CRC from normal controls showed better than that of serum CEA (AUC=0.818) or CA19-9 (AUC=0.650) concentration. This is the first study to report RBP4 and THBS2 as diagnosis serum biomarkers for CRC, which might be a good supplement for CEA or CA19-9 for clinical diagnosis.

## INTRODUCTION

Colorectal cancer (CRC) is the third most common cancer and the fourth leading cause of deaths. It is responsible for over 500,000 deaths annually worldwide [1]. The overall five year survival rate of patients with CRC is 66%. Lots of the evidence suggest that about 90% of the CRC patients who are detected at an early stage can be cured by effective surgical operation and chemo-radio therapy. However, unfortunately, more than 63% CRC patients are diagnosed at an advanced stage, and the survival rate of them is only about 10%-30% [2]. The early diagnostic rate is no more than 37%. Furthermore, common imageological diagnosis cannot improve survival rate limited by its hysteresis [3]. CEA and CA19-9 are currently most common used serum tumor markers for diagnosis of CRC. In CRC patients, CEA and CA19-9 showed various degrees of sensitivity depending on the stage of disease. CEA showed 33% sensitivity and CA19-9 showed only 11% sensitivity at stage II [4]. Since most stage II CRC are potentially curable, the most beneficial diagnostic markers for screening would be able to detect the disease at stage II or even more earlier. Ideal diagnostic markers must be characterized by both high sensitivity and high specificity, CEA and CA19-9 do not show satisfactory sensitivity as diagnostic markers in CRC. Thus finding new serum markers for diagnosis of CRC is necessary.

Over the past decade, obesity has been demonstrated as an independent predictor of incident cancers [5, 6]. The association of several adipokines with common obesity-related cancers has been increasingly recognized. To date, more than 15 adipokines have been reported in the literature [7]. While the circulating levels of majority of pro-inflammatory adipokine levels, such as tumour necrosis factor alpha (TNF-a), IL-6 and leptin are increased in cancers, some adipokines such as adiponectin are protective against tumourigenesis and its serum levels are usually decreased in the patients with cancer. Among these adipokines, RBP4 is synthesized in the liver where it binds vitamin A, retinol, and transports it to tissues throughout the body. It has been implicated as a mediator in the development of insulin resistance and the metabolic disease. Adipose tissue serves as another site of RBP4 synthesis, accounting for its designation as an adipokine. Available information suggests the possibility that RBP4 may be a link between obesity and cancer [8]. But until now, the relationship between RPB4 and colorectal cancer remain unclear.

The thrombospondins (THBSs), a family of five proteins, function in a wide range of settings that involve tissue remodeling, including angiogenesis and neoplasia. They can be divided into two groups on the basis of their molecular architecture since the type 1 repeats (TSRs) are present in THBS1 and THBS2, but not in the other family members (THBS3, THBS4, and THBS5) [9]. The TSRs of THBS1 and THBS2, as well as other proteins, have been shown to mediate their antiangiogenic activity. In 1990, Noel Bouck's laboratory identified THBS1 as the first natural protein inhibitor of angiogenesis [10]. From then, the role of THBS1 as an inhibitor of angiogenesis and tumor progression and the use of THBS-based therapies to inhibit tumor growth has been reported [11–14]. Multiple and varied mechanisms are involved in the inhibition of angiogenesis by THBS1. The TSRs of THBS1 and THBS2 interact with the endothelial cell membrane protein CD36 to inhibit migration and induce apoptosis [15]. THBS1 located in the tumor milieu also function as a suppressor of tumor cell growth by activating TGF-β1 in tumor cells retain the ability to respond to this cytokine [16]. TGF-β conversion from a latent form to a biologically active form is an important step in controlling its functions both *in vitro* and *in vivo*. THBS1 is one of only a few proteins that are able to activate this cytokine *in vivo* [17]. WSHWSPW and RFK sequences in the TSRs are involved in TGF-β binding and activation [18, 19]. THBS2, like THBS1, has been shown to inhibit the angiogenic activity of bFGF in a corneal assay [20] and mitogenesis and formation of focal adhesions in bovine aortic endothelial cells [21, 22]. It also mediates cell-to-cell and cell-to-matrix interactions and is a potent inhibitor of angiogenesis and tumor growth [23]. However, THBS2 function is still largely unknown.

In this study, we investigated in large sample using quantitative ELISA method to explore whether serum RBP4 and THBS2 can act as biomarkers for CRC diagnosis.

## RESULTS

### Serum RBP4 and THBS2 concentrations in CRC patients and normal controls

**Figure 1:**
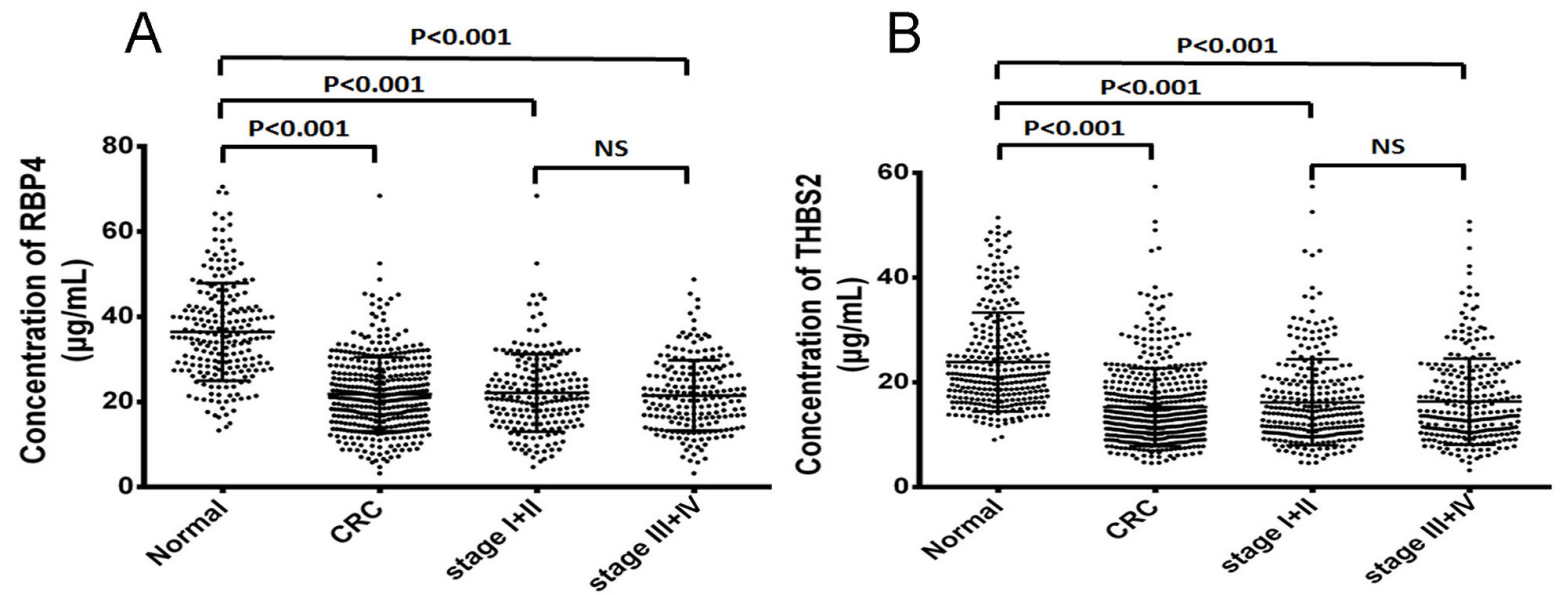
The serum RBP4 and THBS2 concentrations in CRC patients and normal controls **(A)** The serum concentrations of RBP4 was significantly decreased in CRC patients, whether primary or advanced stage. **(B)** The serum concentrations of THBS2 was also significantly decreased in CRC patients, whether primary or advanced stage.

Firstly, RBP4 and THBS2 concentrations in serum from CRC patients and normal controls were evaluated using R&D quantitative ELISA kit. As shown in Figure 1, the mean RBP4 concentration in normal controls were significantly higher than in CRC patients (36.5±11.4 μg/mL vs 21.8±8.7μg/mL, p<0.001). And similar result was found in THBS2 concentration (20.5±6.1 ng/mL vs 14.5±7.3 ng/mL, p<0.001). Both of the serum RBP4 and THBS2 concentrations were significantly decreased in CRC patients. They have the potential ability to distinguish CRC patients from healthy people.

Then, the serum RBP4 and THBS2 concentrations were graded according to the 7th edition of the International Union against Cancer (UICC) tumor node metastasis (TNM). To evaluate whether serum RBP4 and THBS2 concentrations are useful for early detection of CRC, serum concentrations of patients with early stage (stages I+II) were compared with those of normal controls. As shown in Figure 1, the mean concentration of RBP4 or THBS2 had significant difference between early stage CRC patients and normal controls (RBP4: stage I+II vs normal, p<0.001; THBS2: stage I+II vs normal, p<0.001). While no significant difference between stage I+II and stage III+IV patients was found, suggesting that the concentration of RBP4 or THBS2 may not be the proper markers for predicting progression of CRC. All above results indicated that RBP4 or THBS2 can be used as an early diagnosis biomarkers, which provided a new strategy to discriminate CRC patients before they progressed.

### Correlationship between the serum RBP4 and THBS2 concentrations and the clinicopathological features of CRC patients

We analyzed correlationship beteen the serum RBP4 and THBS2 concentrations and the clinicopathologic features of CRC patients. As shown in Table 1, male had significantly higher serum levels of RBP4 than female in both normal controls and CRC patients (p<0.001). The serum RBP4 concentrations in male normal controls and male CRC patients are 42.1±10.5 μg/mL and 23.2±8.2 μg/mL, while in female normal controls and CRC patients are 29.6±8.3 μg/mL and 19.5±8.0 μg/mL, respectively. Besides, we also found CRC patients with drinking habits had relatively higher serum concentration of RBP4 than those avoided alcohol (p=0.034). While no other differences were shown in serum RBP4 concentrations when stratified by TNM stage, tumor metastasis, smoking status, alcohol status, diabetes and Body Mass Index (BMI). As for THBS2, no correlations were found between serum THBS2 concentrations and all clinicopathological characteristics.

**Table 1:** Clinicopathologic features and distribution in the serum concentrations of RBP4 and THBS2

		RBP4 (μg/mL)			THBS2 (ng/mL)	
		n	Mean±SD	*p* Value^a^	Mean±SD	*p* Value^a^
**Normal**						
**Gender**				<0.001^*^		0.174
	Male	108	42.1±10.5		20.2±7.3	
	Female	108	29.6±8.3		20.7±4.1	
**CRC patients**						
**Gender**				<0.001^*^		0.971
	Male	248	23.2±8.2		14.8±9.8	
	Female	154	19.5±8.0		14.5±7.6	
**TNM Stage**				0.612		0.751
	I-II	207	22.1±9.1		14.3±7.1	
	III- IV	195	21.5±8.3		15.2±10.7	
**Tumor Metastasis**				0.943		0.184
	Metastasis	71	22.0±9.2		15.8±9.0	0.249
	Non-metastasis	331	21.7±8.5		14.2±6.9	
**Smoking**				0.063		0.125
	Yes	136	21.9±9.5		15.0±8.1	
	No	266	23.8±7.7		13.4±5.9	
**Alcohol**				0.034^*^		0.304
	Yes	127	24.2±8.5		14.8±8.1	
	No	275	21.7±9.1		13.8±6.2	
**Diabetes**				0.541		0.309
	Yes	35	22.4±7.5		16.0±8.1	
	No	367	21.8±8.8		14.6±9.1	
**BMI**				0.066		0.813
	<18.5	39	18.9±7.4		14.8±7.5	
	18.5-25	266	21.7±8.4		14.7±9.9	
	>25	97	23.4±9.8		14.5±7.2	
**Chronic diarrhea or constipation**				0.846		0.408
	Yes	199	21.8±8.3		14.7±7.4	
	No	203	21.9±9.1		14.8±10.5	
**cholecystectomy or appendicectomy**				0.851		0.499
		50	21.4±8.2		15.2±8.6	
		352	21.9±8.8		14.7±9.1	
**CRC in family or HAP**				0.832		0.558
	Yes	65	21.2±8.4		14.8±8.0	
	No	98	21.0±7.7		15.1±12.7	

^a^Mann-Whitney U (two-sided test).

^*^p values were statistically significant.

In addition, we also compared the the serum RBP4 and THBS2 concentrations and some high risk factors of CRC according to the definition by American Cancer Society [24] including the history of chronic diarrhea or constipation, the history of cholecystectomy or appendectomy and the history of CRC in family or Familial Adenomatous Polyps (FAP). However, no correlations were found between serum RBP4 or THBS2 concentrations and these high risk factors of CRC.

**Figure 2:**
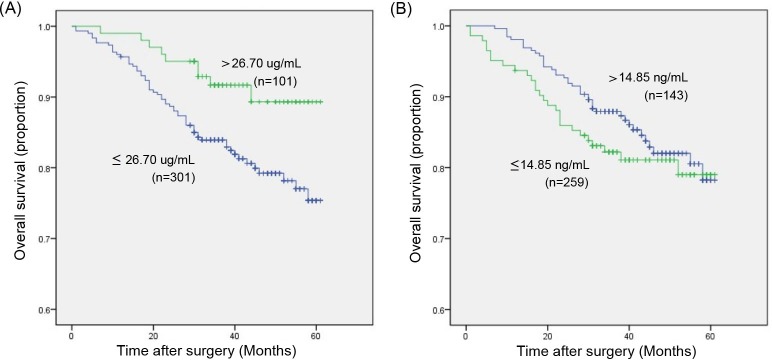
Kaplan-Meier survival curves of CRC patients according to serum RBP4 or THBS2 concentrations **(A)** The overall survival rate for patients with high serum RBP4 concentrations (>26.70 ug/mL) was significantly higher than that for patients with low serum RBP4 concentrations (≤26.70 ug/mL). **(B)** No significant difference between the overall survival rate for patients with high serum THBS2 concentrations (>14.85 ng/mL) and that for patients with low serum THBS2 concentrations (≤14.85 ng/mL).

To determine the association between serum RBP4 or THBS2 concentrations and prognosis of colorectal cancer patients, all patients were followed-up for overall survival after surgery. Kaplan-Meier survival (Figure 2) indicated that the overall survival rate for patients with high serum RBP4 concentrations (>26.7 ug/mL) was significantly higher than that for patients with low serum RBP4 concentrations (≤26.7 ug/mL), while there was no significant difference between the overall survival rate for patients with high serum THBS2 concentrations (>14.85 ng/mL) and that for patients with low serum THBS2 concentrations (≤14.85 ng/mL). Univariate analysis showed that TNM stages (P<0.001), tumor metastasis (P<0.001), diabetes (p=0.049), BMI (P=0.030), iFOBT (p=0.012) and RBP4 (p=0.022) associated with overall survival rates (Table 2). Therefore, multivariate analysis was performed depending on the Cox proportional hazards model for the variables with p-value <0.05 examined in the univariate analysis. After excluding TNM stages, BMI and iFOBT by forward LR method, we found that tumor metastasis (HR: 4.375; 95% CI: 2.315-8.267; p<0.001), diabetes (HR: 2.514; 95% CI: 1.264-5.000; p=0.009) and RBP4 (HR: 0.409; 95% CI: 0.200-0.837; p=0.014) proved to be independent prognostic factors for survival in colorectal cancer (Table 2). According to these data above, we have a preliminary conclusion that serum RBP4 concentrations could be a valuable prognostic factor in colorectal cancer, while THBS2 could not.

**Table 2:** Univariate and multivariate Cox analyses between various factors contributing to the survival of CRC patients

Variables	Univariate			Multivariate		
	HR	95% CI	*p* Value	HR	95% CI	*p* Value
Gender (male/female)	1.072	0.659-1.743	0.779			
TNM Stage (I+II/III+IV)	3.500	2.021-6.062	**<0.001**	1.614	0.786-3.314	0.192
Tumor Metastasis (yes/no)	5.569	3.459-8.966	**<0.001**	4.375	2.315-8.267	**<0.001**
Smoking (yes/no)	0.799	0.476-1.339	0.393			
Alcohol (yes/no)	0.914	0.556-1.503	0.724			
Diabetes (yes/no)	1.964	1.003-3.846	**0.049**	2.514	1.264-5.000	**0.009**
BMI (<18.5,18.5-25,>25)	0.604	0.382-0.953	**0.030**	0.677	0.433-1.060	0.088
iFOBT (positive/negative)	0.541	0.336-0.872	**0.012**	0.636	0.381-1.063	0.084
Chronic diarrhea or constipation (yes/no)	0.890	0.552-1.432	0.630			
cholecystectomy or appendicectomy (yes/no)	0.614	0.265-1.421	0.254			
History of CRC or HAP (yes/no)	0.427	0.172-1.058	0.066			
RBP4 (≥26.7 vs <26.7)	0.441	0.219-0.890	**0.022**	0.409	0.200-0.837	**0.014**
THBS2 (>14.85 vs ≤14.85)	1.261	0.776-2.050	0.349			

### Performance of RBP4 and THBS2 in the detection of CRC patients

**Figure 3:**
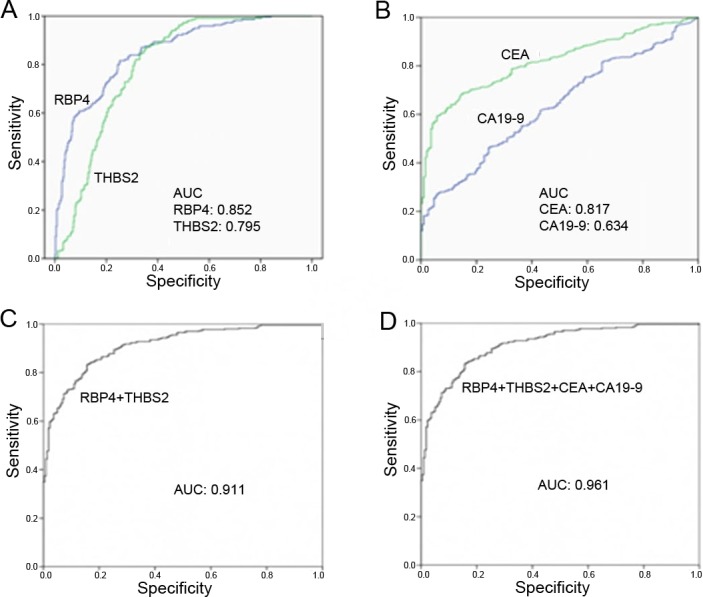
ROC curve analysis of serum concentrations from patients with CRC and controls **(A)** The AUC performance of RBP4 and THBS2 respectively. **(B)** The AUC performance of CEA and CA19-9 respectively. **(C)** The AUC performance of combination use of RBP4 and THBS2. **(D)** The AUC performance of combination use of RBP4, THBS2, CEA and CA19-9.

To further detect the distinguish performance, ROC curves were developed in Figure 3. RBP4 predicted the diagnosis of CRC patients with an AUC of 0.853 (95% CI: 0.822-0.883) at a cutoff point of 26.70 μg/mL. This cutoff point provided 74.9% sensitivity and 81.7% specificity. Meanwhile, THBS2 differentiated CRC from normal controls with 64.6% sensitivity and 87.1% specificity at a cutoff point of 14.85ng/mL. AUC was 0.794 (95% CI: 0.759-0.828).

The odds ratios (OR) were used to describe the predictive value of serum RBP4 and THBS2 to CRC as shown in Table 3. The OR was 0.230 (95% CI: 0.180-0.294, p<0.001) per 10μg/mL decrease in RBP4 before adjusted. When it was adjusted by gender and alcohol, the OR was 0.151 (95% CI: 0.105-0.215, p<0.001) per 10μg/mL decrease. THBS2 showed statistically significant decreased OR with 0.294 (95% CI: 0.221-0.394, p<0.001) per 10 ng/mL.

**Table 3:** Logistic regression analysis of potential markers and clinical markers

	Unadjusted			Adjusted by gender and alcohol		
	OR	95% CI	*p* Value	OR	95% CI	*p* Value
RBP4 (per 10μg/mL)	0.230	0.180-0.294	**<0.001**	0.151	0.105-0.215	**<0.001**
THBS (per 10 ng/mL)	0.294	0.221-0.394	**<0.001**			
CEA (per 1ng/mL)	2.071	1.734-2.474	**<0.001**			
CA19-9 (per 10U/mL)	1.677	1.373-2.049	**<0.001**			

Abbreviations: OR, odds ratio, CI, confidence interval.

Currently, CEA and CA19-9 are commonly used as serum biomarkers for blood-based CRC screening. To evaluate the value of RBP4 and THBS2 in clinical application, we contrasted to biomarkers of CEA and CA19-9. We also detected serum levels of CEA and CA19-9 by ELISA assay. In this control and case assay, the AUC for the ability of CEA to predicts the CRC patients from normal was 0.817 (95% CI: 0.784-0.851) and the OR of CEA is 2.071 (95% CI: 1.734-2.474, p<0.001) per 1 ng/mL increased. While, CA19-9 had relatively low AUC value 0.634 (95% CI: 0.587-0.678), and its OR is 1.677 (95% CI: 1.373-2.049, p<0.001) per 10 U/mL increased. We can see that the ability of RBP4 used as diagnosis CRC marker seemed superior to THBS2 and the combined discrimination ability of RBP4 and THBS2 was better than CEA or CA19-9.

### Combination of RBP4 or THBS2 with other clinical biomarkers to improve diagnosis value in CRC patients

Combination of RBP4 or THBS2 with clinical markers was able to effectively differing CRC patients which significantly improved the specificity and sensitivity. As shown in Table 4, combination of RBP4 and CEA demonstrated a higher AUC (0.927, 95% CI: 0.906-0.947) with sensitivity of 80.8% and specificity of 91.2%. The simultaneous use of RBP4 and CA19-9 gave an AUC (0.856, 95% CI: 0.825-0.888) with sensitivity of 74.9% and specificity of 81.7%. The combination application showed better clinical diagnostic efficacy than that of CEA or CA19-9 used alone. By contrast, the sensitivities of CEA or CA19-9 used alone were much lower, 68.3% and 45.6%, respectively.

**Table 4:** Diagnostic performance of independent or combinations of makers

	AUC (95% CI)	Sensitivity (%)	Specificity (%)	cutoff value
RBP4	0.853(0.822-0.883)	74.9	81.7	26.70
THBS2	0.794(0.759-0.828)	64.6	87.1	14.85
CEA	0.817(0.784-0.851)	68.3	85.5	2.51
CA19-9	0.634(0.587-0.678)	45.6	75.6	12.6
RBP4+CEA	0.927(0.906-0.947)	80.8	91.2	
RBP4+CA19-9	0.856(0.825-0.888)	74.9	81.7	
RBP4+CA19-9+CEA	0.927(0.907-0.948)	79.7	91.7	
THBS2+CEA	0.897(0.871-0.923)	77.2	89.6	
THBS2+ CA19-9	0.838(0.806-0.870)	65.7	93.2	
THBS2+ CA19-9+CEA	0.902(0.877-0.927)	79.9	89.6	
RBP4+THBS2	0.911(0.888-0.933)	83.3	84.3	
RBP4+THBS2+CA19-9+CEA	0.961(0.947-0.975)	87.1	92.7	

Sensitivity, specificity and predicted optimal cutoff value of the combinational diagnosis from the maximum Youden's index.

As RBP4 performance, combination use of THBS2 and clinical marker can also enhance the ability to detection of CRC. The AUC, sensitivity and specificity can be further improved to the highest when these four maker are used together. Considering the cost and convenience, combine RBP4 and CEA maybe the most appropriate strategy of diagnosis of CRC.

## DISCUSSION

Patients with advanced stage CRC usually have a poor prognosis and a high rate of mortality. Successful treatment of CRC, which depends largely on early accurate diagnosis, is the key to improve survival rate and life qualities of patients. The gold standard in detecting CRC even high-risk adenomas is still colonoscopy which is expensive especially the painless colonoscopy. But its invasiveness, the experience of discomfort, the potential risks of complications, and the resources needed for the screening itself are disadvantages of concern [25, 26]. Serum proteins and other components like genetic biomarkers such as loss of heterozygosity, mutation and epigenetic biomarkers such as DNA methylation, histone modification and non-coding RNAs can serve as convenient and inexpensive biomarkers of diseases, and are expected to play important roles in the diagnosis of early stage CRC [27–30]. In this study we evaluated RBP4 and THBS2 as two novel serum biomarkers for CRC diagnosis and showed that their performance were better than that of CEA or CA19-9. Combined use of these four markers can further significantly improve the diagnosis of CRC.

RBP4 belongs to the lipocalin family and is the specific carrier for retinol in the blood. RBP4 is an adipocyte-secreted molecule that is elevated in the serum seems to signal the presence of insulin resistance and associated cardiovascular risk factors [31]. Serum RBP4 level is often used as a clinical indicator of kidney disease for early diagnosis and curative effect evaluation [32, 33]. Until now, study results of serum RBP4 concentration in cancers are uncertain. Though some studies showed that serum RBP4 was upregulated in some cancers such as ovarian and pancreatic cancers [34, 35], many other studies found significantly decreased levels of serum RBP4 in cancers such as ovarian cancer [36], HCC [37, 38], head and neck cancer [39], and breast cancer [40]. While the reason of downregulation may due to the methylation [41, 42]. The adipocyte participates as a central mediator of innate immune response, in which adipokines secretion is responsible for a paracrine loop between adipocytes and macrophages. This interplay would contribute to low-grade inflammation, which provided a favorable niche for tumor development [43]. Adipokines remain one of the major players in obesity-related carcinogenesis. To date, more than 15 adipokines have been reported to be associated with cancers [44]. Among these, the circulating levels of majority of pro-inflammatory adipokines, such as leptin, IL-6 and tumour necrosis factor alpha (TNF-a) are increased in cancers, while some adipokines such as adiponectin are protective against tumourigenesis and its serum levels are usually decreased in the patients with cancer [45]. A meta-analysis have suggested a negative association of leptin or adiponectin, positive associations of resistin with CRC [46, 47]. RBP4 as one of adipokines, may play an important role in reducing immune response and inflammation. RBP4 might provide a new direction for cancer biomarker research, which needs to be confirmed by much more studies. We noticed that recently a report associated colon adenoma risk with high circulating levels of RBP4 [48]. The result seems inconsistent with our results. Colorectal cancer developed through a progressive process from normal mucosa to benign adenoma and then to carcinoma [49–51]. Although benign, adenomas are the direct precursors of adenocarcinomas and follow a predictable cancerous temporal course unless interrupted by treatment. They are divided into three subtypes based on histologic criteria, as follows: (1) tubular, (2) serrated, and (3) villous. Villous adenomas are of concern because of their higher risk of malignant transformation than tubular and serrated adenomas. So we think if the authors could analyze the correlationship of serum RBP4 in each adenoma subgroup, the results are more meaningful.

Several studies reported that serum RBP4 concentrations correlate with sex, and the concentration in men is higher than that in women [52, 53], we also found the serum level of RBP4 was correlated to gender. Similarly, alcohol drinking can decrease the serum retinol concentration in head and neck cancer [54]. But until now, there have no information imply the relationship between alcohol and serum RBP4 levels in CRC. In this study, we first report that serum RBP4 level increased in alcohol drinking CRC patients. These two clinicopathologic features as interference factors eliminated in adjusted OR model. Since follow-up visit data were limited, we could not further analyze whether this two markers can be prognosis factors.

THBS2 is a member of thrombospondin family proteins. It is usually considered as an endogenous negative regulator of angiogenesis in tumorigenesis [55]. In many tumors, down-regulation of thrombospondins accompanies activation of oncogenes or inactivation of tumor suppresser genes. Increasing the expression levels of THBS1 or THBS2 in tumor tissue can inhibit tumor growth [56–60]. Expression of THBS2 in CRC is associated with the inhibition of angiogenesis and a reduced frequency of distant metastasis [58]. Bein K et al have reported that the mechanism for the inhibition of tumor growth by THBS1 involves the inhibition of MMP9 mediated mobilization of VEGF [61]. Similar to THBS1, THBS2 also binds to MMP9, indicating that it has a similar function. N-terminal recombinant fragment of THBS2 inhibited breast cancer growth and metastasis by CD36 mediated activation of endothelial cell apoptosis [62]. THBS2 mediates cell-to-cell and cell-to-matrix interactions and may function as either a potent inhibitor [63–65] of tumor growth and angiogenesis in ovarian carcinoma. Recently, Slavin S et al studied the role of cancer-associatedfibroblasts (CAF) estrogen receptor alpha (ERα) and found that it could protect against prostate cancer invasion. ERα could function through a CAF-epithelial interaction via selectively upregulating THBS2. Knockdown of THBS2 led to increased MMP3 expression and interruption of the ERα mediated invasion suppression, providing further evidence of an ERα-THBS2-MMP3 axis in CAF [66]. Moreover, Salvianolic acid B (Sal B) has an inhibitory effect on oral squamous cell carcinoma cell growth. The antitumor effect can be attributed to antiangiogenic potential induced by a decreased expression of some key regulator genes of angiogenesis while expression of THBS2 was up-regulated [67]. These high THBS2 promoter methylated in blood were reported in the recurrent endometrial adenocarcinoma patients and THBS2 had methylation in the primary tumor as well [68]. It is also known to be methylated in a variable number of breast neoplasms [69]. Most recently, microRNA-135b (miR-135b), a key regulator of the malignancy, was reported to be highly expressed in the RC component and promoting MLS cell invasion *in vitro* and metastasis *in vivo* through the direct suppression of THBS2. Though recently, Wang X et al reported that THBS2 expressed significantly higher in CRC tissue when compared with paired adjacent normal tissue. We noticed that they just analyzed mRNA expression but not serum THBS2 protein expression [70].

In conclusion, to the best of our knowledge, this is the first study to report RBP4 and THBS2 as diagnosis serum biomarkers for CRC, which might be a good supplement for CEA or CA19-9 for clinical diagnosis. Further mechanism study of these two proteins is needed to reveal the principles of the difference serum level between normal and tumor.

## MATERIALS AND METHODS

### Patients and serum samples

CRC Serum samples were obtained with informed consent before surgical resections from Sir Run Run Shaw Hospital, Zhejiang University, Hangzhou, China. This retrospective study includes 402 CRC serum samples and 218 normal samples between 2013 and 2015. CRC patients were histopathologically confirmed by pathology department. Patients with CRC in the study never received preoperative radiotherapy, chemotherapy, or chemoradiotherapy. Those who had inflammatory diseases, including infections, ischemic heart disease, collagen diseases, or bowel perforation and obstruction, also were excluded. Normal serum samples were collected from health examinations with no clinical evidence of CRC. This study was approved and monitored by the ethics committee of Sir Run Run Shaw Hospital, Zhejiang University.

### Enzyme linked immunosorbent assay

The concentrations of RBP4 and THBS2 were measured in triplicates with ELISA which performed according to the procedure of R&D Systems (Catalog Number: RBP4 DRB400 and THBS2 DTSP20). The LOD of RBP4 Elisa kit DRB400 is 0.224 ng/mL and its detecting range is 1.6-100 ng/mL. The LOD of THBS2 Elisa kit DTSP20 is 0.025 ng/mL and its detecting range is 0.3-20 ng/mL.

Data read by a multi-detection microplate reader (BioTek Synergy 2, USA) set to 450nm and 540nm for correction. Take consider of measurement error among batch, we revised data by standard curve each plate tried to keep the same conditions and external environment.

### CA19-9 and CEA assay

CA19-9 and CEA levels were measured by an automated immunoassay system (Architect i2000; Abbott Diagnostics Division). The working range of the CA19-9 immune assay is 0.5-1200 U/ml, and CEA is 0.4-1500ng/mL.

### Statistical analysis

Statistical analysis was carried out using the SPSS 22.0 software package (SPSS, IBM, Chicago, IL, USA). All quantitative variables are expressed as means standard deviations unless stated otherwise. Univariate comparisons between groups (cases and controls) were performed using chi-square tests or Fisher exact tests for categorical data, and using Mann-Whitney *U* test for continuous variables.

A receiver operating characteristic (ROC) curve was plotted to assess diagnostic performance. The area under the curve (AUC) and 95% confidence interval were used to assess the discriminatory power. Combination diagnosis was performed to create a new parameter by using a logistic regression model, and obtain new ROC and AUC values for combined biomarkers. Conditional logistic regression models were used for estimating odds ratios and 95% confidence intervals to evaluate the association of each variable with CRC. Correlation analyses were performed based on the characteristics of variables. A p<0.05 was considered statistically significant.
